# Relaparoscopy in the management of post-operative complications after minimally invasive gastrectomy for gastric cancer

**DOI:** 10.1007/s13304-022-01328-z

**Published:** 2022-07-26

**Authors:** Ugo Elmore, Marco Milone, Paolo Parise, Nunzio Velotti, Andrea Cossu, Francesco Puccetti, Lavinia Barbieri, Sara Vertaldi, Francesco Milone, Giovanni Domenico De Palma, Riccardo Rosati

**Affiliations:** 1 Department of Gastrointestinal Surgery, San Raffaele Hospital, Milan, Italy; 2 Department of Clinical Medicine and Surgery, University of Naples Federico II, Via Pansini n.5, 80131 Naples, Italy; 3 Department of Advanced Biomedical Sciences, University of Naples Federico II, Naples, Italy

**Keywords:** Relaparoscopy, Gastric cancer, Minimally invasive surgery

## Abstract

Laparoscopy has already been validated for treatment of early gastric cancer. Despite that, no data have been published about the possibility of a minimally invasive approach to surgical complications after primary laparoscopic surgery. In this multicentre study, we describe our experience in the management of complications following laparoscopic gastrectomy for gastric cancer. A chart review has been performed over data from 781 patients who underwent elective gastrectomy for gastric cancer between January 1996 and July 2020 in two high referral department of gastric surgery. A fully descriptive analysis was performed, considering all the demographic characteristics of patients, the type of primary procedure and the type of complication which required reoperation. Moreover, a logistic regression was designed to investigate if either the patients or the primary surgery characteristics could affect conversion rate during relaparoscopy. Fifty-one patients underwent reintervention after elective laparoscopic gastric surgery. Among patients who received a laparoscopic reintervention, 11 patients (34.3%) required a conversion to open surgery. Recovery outcomes were significantly better in patients who completed the reoperation through laparoscopy. Relaparoscopy is safe and effective for management of complications following laparoscopic gastric surgery and represent a useful tool both for re-exploration and treatment, in expert and skilled hands.

## Introduction

Laparoscopy has already been validated for treatment of early gastric cancer with an advantage in short-term recovery outcomes and good oncological results also in terms of disease-free survival rate. [1, 2]. It was also proposed as a valid approach in advanced carcinoma as shown in the “Stomach trial”. [3].

Despite that, no data have been published about the possibility of a minimally invasive approach to surgical complications after primary laparoscopic surgery. This approach is challenging, not always indicated, and requires experienced laparoscopic surgeons. For these reasons, laparoscopy for the treatment of complications after gastric surgery is not commonly applied.

In this multicentre study, we described our experience in the management of complications following laparoscopic gastrectomy for gastric cancer and evaluated feasibility, safety and efficacy of a re-laparoscopic (RL) approach.

## Materials and methods

### Study setting

A chart review has been performed over data from a total of 781 patients who underwent elective gastrectomy for gastric cancer between January 1996 and July 2020 in two high referral departments of gastric surgery. Of these, 295 received a laparoscopic gastrectomy and 51 (17.2%) required surgical revision for peri-operative complications. In 32 cases, a re-laparoscopy was performed while in 19 cases, due to comorbidities and haemodynamic instability of patients, an open approach was chosen.

Patients data were extracted from the surgical reports of the two centres and demographic and surgical characteristics were obtained; in detail, gender, age, body mass index (BMI), American Society of Anesthesiologists (ASA) score, previous abdominal surgery, type of primary procedure, type of complication which required reoperation, characteristics of reintervention, conversion to open surgery, operative time, grade of complications according to Clavien–Dindo classification [4] and hospital stay (from re-laparoscopy to discharge) were retrospectively reviewed.

### Surgical management of complications

In case of anastomotic leakage, the operative strategy took into account the degree of peritoneal contamination and the status of the anastomosis: for small leaks, the repair of the anastomosis was the treatment of choice, but in case of extended peritoneal contamination or/and a large anastomotic defect, anastomosis re-do was preferred. Feeding jejunostomy was realized selectively. For internal/incisional hernia with small bowel obstruction, reduction of the herniated bowel and suture of the hernia defect was obtained. In case of small bowel resection, a side-to-side mechanical anastomosis was performed. In cases of bleeding, haemostasis was achieved with different methods: bipolar coagulation, placement of clips, sutures or local haemostatic agents.

### Data analysis

A fully descriptive analysis was performed over demographic characteristics of patients (age, gender, BMI, ASA score and previous abdominal surgery), the type of primary procedure and the complications which required reoperation. Operative time, kind of procedure performed to solve the complication and recovery outcomes were also evaluated.

Moreover, among patients who underwent minimally invasive technique, a logistic regression was designed to investigate if either the patients or the primary surgery characteristics could affect conversion rate during relaparoscopy. A comparison between patients who received a totally laparoscopic reoperation and patients who needed conversion to open surgery during minimally invasive approach was performed.

Statistical analysis was completed with SPSS version 26.0 (IBM, Armonk, NY). Continuous variables are described as means ± standard deviation and compared by the Mann–Whitney *U*-test; categorical variables are reported as percentages and compared by the *χ*^2^ test; the Wald test was used to assess the significance of logistic regression. A *p* value of less than 0.05 was defined as statistically significant.

## Results

Fifty-one out of 295 patients underwent reintervention after elective laparoscopic gastric surgery.

There were 24 male patients and 27 female patients, mean age was 67.29 ± 13.03 years, mean BMI was of 24.69 ± 5.41 kg/m^2^ and mean ASA score was of 2.3 ± 0.46. Twenty-nine patients had received previous abdominal surgery: tumour was located in 313 cases at the antrum (8 cases at the angulus), in 17 cases at the corpus, and in 3 cases at the fundus. Primary surgery was a subtotal gastrectomy in 25 cases and a total gastrectomy in 26 cases.

Thirty-two patients received a re-laparoscopy and 19 patients an open approach. About indication to reintervention, in the laparoscopic group, we recorded: nine anastomotic leaks, one anastomotic stenosis, ten bleedings, three duodenal leak, one incisional hernia, two small bowel obstruction, one pancreatic fistula and five bowel perforations. Indication to reoperation in the open approach group was: seven anastomotic leaks, eight bleedings, one duodenal leak, one small bowel obstruction and two bowel perforations.

As for the specific operative strategy: in case of anastomotic leaks, in three cases a new anastomosis was fashioned, in 1 case the anastomosis was repaired and in 5 cases a conversion to open surgery was necessary; in case of stenosis, the anastomosis was re-fashioned; in case of bleeding, in seven cases the haemostasis was achieved laparoscopically with coagulation, clips and use of haemostatic agents, but in three cases a conversion to open surgery was necessary; in case of duodenal leak, in one patient a feeding jejunostomy was realized and in one patient a duodenal suture with Kehr tube placement was performed but conversion was needed; in case of incisional hernia, which occurred on the Pfannenstiel incision, it was managed laparoscopically with bowel reduction and then direct repair; in case of intestinal obstruction a laparoscopic small bowel resection was performed in 1 case and conversion to open surgery in the other case; in case of bowel perforations, 2 patients underwent direct suture and 2 patients underwent bowel resection and anastomosis; finally, the only case of pancreatic fistula, required the totalization of the gastrectomy with an oesophago-jejunal anastomosis after conversion to open surgery.

Among patients who received a laparoscopic reintervention, 11 patients (34.3%) required a conversion to open surgery. Primary surgery was total gastrectomy in 5 patients and subtotal gastrectomy in 6 patients; in most cases, the tumour was located at the antrum (45.4%). Between conversion and fully laparoscopic groups, no differences were found in terms of gender (11/21 vs 5/11 males, *p* = 0.8), mean age (69.61 ± 11.31 vs 65.56 ± 14.09, *p* = 0.3) and ASA score (2.30 ± 0.48 vs 2.28 ± 0.46, *p* = 0.9). Operative time was longer in the conversion group (166.9 ± 36.03 vs 93.9 ± 25.68, *p* = 0.001); recovery outcome, such as Clavien–Dindo score (*p* = 0.01), and hospital stay (37.63 ± 20.83 vs 20.81 ± 11.93, *p* = 0.02), were significantly better in patients who completed the reoperation through laparoscopy. Patients’ data are presented in Table 1.

**Table 1:** Patients’ characteristics

Characteristics	All laparoscopic patients (*n* = 32)	Fully laparoscopic (*n* = 22)	Conversion (*n* = 11)	*p*-value
Male (*n*, %)	16/32 (50%)	11/22 (50%)	5/11 (45.4%)	0.8
Age (mean ± SD)	66.95 ± 13.16	65.56 ± 14.09	69.61 ± 11.31	0.38
BMI (mean ± SD)	24.84 ± 6.47	24.39 ± 5.92	25.69 ± 7.65	0.62
ASA score (mean ± SD)	2.29 ± 0.46	2.28 ± 0.46	2.30 ± 0.48	0.9
Previous abdominal surgery (*n*)	18/32 (56.2%)	10/22 (45.4%)	8/11 (72.7%)	0.13
Tumour localization (*n*)	–	–	–	0.21
Antrum	16	12	11	–
Corpus	8	10	11	–
Fundus	2	4	4	–
Angulus	6	4	–	–
Type of gastrectomy (*n*)	–	–	–	0.81
Total	15	10	5	
Subtotal	17	12	5	
Operative time (mean ± SD)	117.45 ± 45.09	93.9 ± 25.68	166.9 ± 36.03	0.001
Clavien–Dindo (mean ± SD)	3.48 ± 0.72	3.25 ± 0.55	3.91 ± 0.83	0.03
Hospital stay (mean ± SD)	26.5 ± 17.19	20.81 ± 11.93	37.63 ± 20.83	0.02

**Fig. 1:**
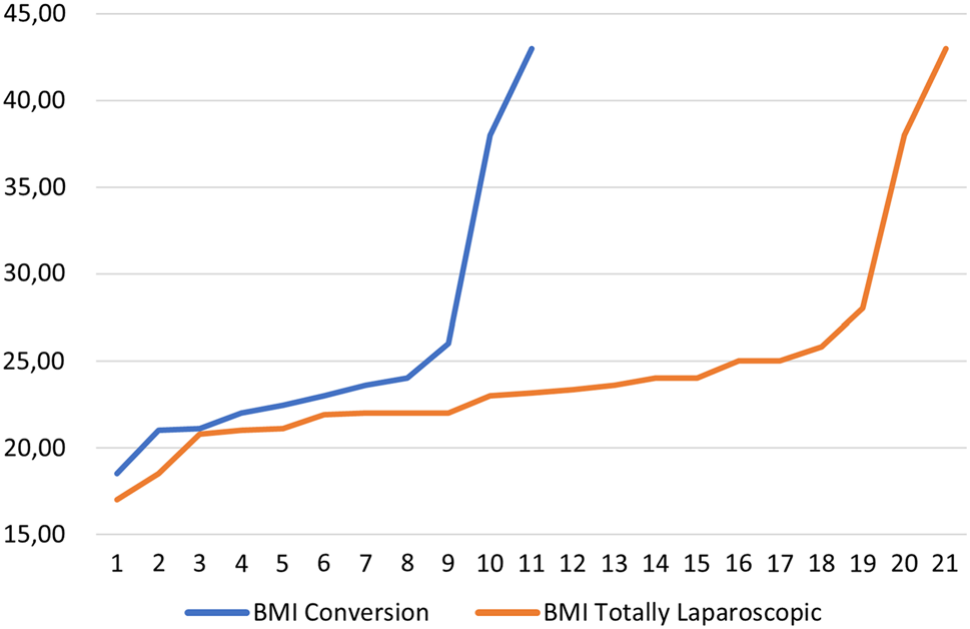
BMI distribution among patients who received reintervention with a totally laparoscopic approach and patients who required conversion to open surgery

**Fig. 2:**
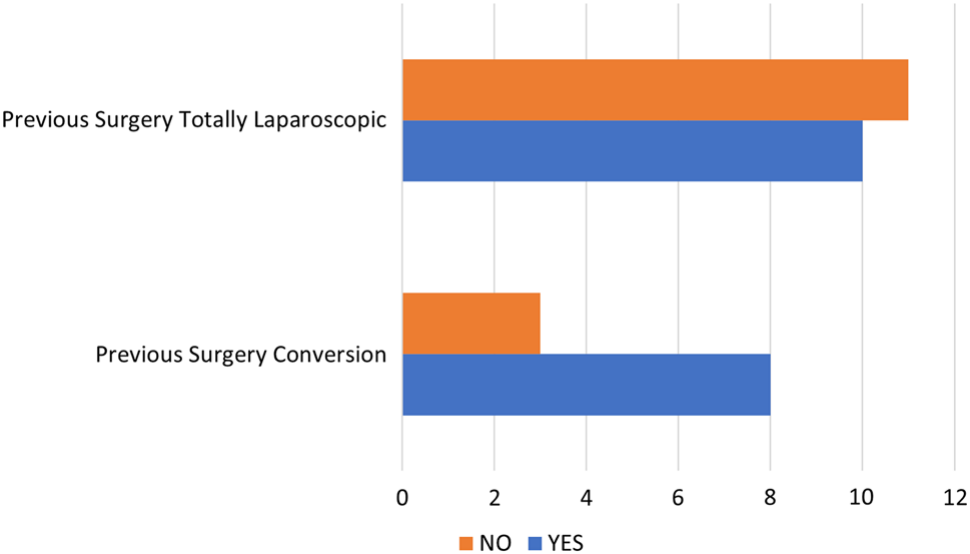
Previous abdominal surgery rate between patients who received reintervention with a totally laparoscopic approach and patients who required conversion to open surgery

Logistic regression indicated that no predictors (gender, age, BMI, ASA score, previous abdominal surgery and type of gastrectomy performed) significatively impact on conversion rate but the mean BMI was lower in patients who had a fully laparoscopic approach (25.69 ± 7.65 vs 24.39 ± 5.92, *p* = 0.6); similarly, in conversion group there was a higher rate of patients who received a previous abdominal surgery (72.7% vs 45.4%, *p* = 0.13). (Fig. 1,2).

Reasons for conversion during laparoscopic reintervention, were in most cases the inability to find the site of leak/perforation, presence of extended necrosis of the gastric remnant, presence of diffuse adhesions and the biliary contamination of the peritoneal cavity. (Tables 2, 3).

**Table 2:** Laparoscopic surgical management of complications

Complication	*n*	Management
Anastomotic leak	9	3 New fashioned anastomosis / 1 anastomosis repair + **5 conversion**
Anastomotic stenosis	1	Re-fashioned anastomosis
Bleedings	10	7 Haemostasis + **3 conversions**
Duodenal leak	3	2 Nutritional jejunostomy + **1 conversion**
Incisional hernia	1	Hernia repair
Intestinal Occlusion	2	Small bowel resection in 1 case + **1 conversion**
Pancreatic fistula	1	Oesophageal-jejunal anastomosis **(converted)**
Bowel perforation	5	2 Perforation repair/3 bowel resection with anastomosis

Conversions to open surgery are reported in bold

**Table 3:** Conversions to open surgery

Complication	*n*	Management
Anastomotic leak of a Roux—an—Y jejunal anastomosis	1	Realization of jejunostomy and redo a jejunum-jejunal anastomosis
Trans-mesocolic hernia	1	Large small bowel resection for extended ischemia
Acute bleedings	3	Haemostasis of a copious bleeding
Duodenal leak	1	Duodenal suture and T Kehr drainage positioning
Gastro-jejunal leak	2	Extended abdominal contamination required lavage before fashioning a new anastomosis
Necrosis of gastric remnant + spleen injury + pancreatic fistula	1	Complete gastric resection with oesophageal-jejunal anastomosis + splenectomy
Oesophageal-jejunal anastomosis leak	2	Nutritional jejunostomy

## Discussion

After the first description of a laparoscopic-assisted gastrectomy for gastric cancer in 1994 [5], this approach became more and more widespread. Several advantages of the laparoscopic approach have been shown, especially in randomized studies in the East (particularly in Korea, Japan and China) and this method is increasingly evolving as a standard of care [6]. Many studies have confirmed the feasibility, safety and oncologic equivalency compared with open gastrectomy for early and more recently for advanced gastric cancer [7, 8]. A recent European randomized trial in multimodal setting, shows excellent results justifying the use of the laparoscopic approach in the daily practice; in this study, the minimally invasive approach can provide good results in short and medium-term survival without an increase in recurrence and distant metastasis [9, 10].

Less is known about the role of the laparoscopic approach in case of complications after primary laparoscopic surgery. Re-laparoscopy has been successfully used for management of complications after colorectal surgery [11], but there are no studies reporting about this approach in gastric cancer surgery.

Dexter et al. [12] proposed the adoption of the laparoscopic approach to surgical complications describing the results from 13 patients submitted to re-laparoscopy within 7 days of laparoscopic cholecystectomy for intra-abdominal bleeding or abdominal pain. The authors reported good results both in terms of resolution of complications and recovery, concluding that laparotomy can be avoided by prompt re-laparoscopy in patients with abdominal complications of laparoscopic cholecystectomy. Interesting results were described also by Barband [13] and Wills [14] who analysed data from 9 and 10 patients, respectively, who underwent re-laparoscopy for treatment of minor bile leakage after laparoscopic cholecystectomy; they found a success rate of about 90%, concluding that this is an effective procedure in selected situations.

About colorectal surgery, Marano et al. [15] reported of 20 patients submitted to re-laparoscopy for the management of postoperative peritonitis after a primary laparoscopic colorectal intervention; they had a conversion rate of 10% and an overall morbidity of 50% with no 30-day mortality. The authors concluded that, in haemodynamically stable patients, a prompt laparoscopic reoperation is an accurate diagnostic tool and could be an effective and safe surgical option.

Similarly, Cuccurullo et al. [16] in a retrospective study on 84 patients who had re-laparoscopy after laparoscopic colorectal surgery for postoperative complications, found a low morbidity rate and good recovery outcomes, concluding that this approach is a safe and effective tool for management of complications and represents the first step of re-exploration and treatment. Finally, a recent systematic review by Chang et al. [11] on 11 studies reporting laparoscopic re-intervention for complications in 187 patients following laparoscopic colorectal surgery, found a success rate of 96% maintaining the benefits of the laparoscopic approach and avoiding a laparotomy. The authors still underlined that this approach appears to be safe and effective in highly selected patients.

In gastric surgery, laparoscopy is a great technical improvement which provides clear advantages for the patient. Recent literature and studies on laparoscopic gastrectomy showed that the same complications known to affect open gastric surgery are present in the laparoscopic approach. [17–19] On the other hand, no study has ever focused on the possibility and on the possible advantages of managing these complications with a minimally invasive technique. For this reason, we reported about our experience in re-laparoscopy for treatment of early complications following primary laparoscopic gastric surgery.

In the present series, re-intervention was mainly necessary for anastomotic leaks, bleeding and iatrogenic bowel perforations and the success rate of RL was 65.6%. We think that the conversion rate of laparoscopic reoperation is acceptable. Interestingly, we recorded that high BMI and previous abdominal surgery are risk factors for conversion. In effect, obesity is per se a factor related to a higher conversion rate because of the technical difficulties determined by intra-abdominal fat [20, 21]. Similarly, previous abdominal surgery with the related abdominal adhesions could limit laparoscopic approach and may require conversion to open surgery.

The reasons for conversion during laparoscopic re-intervention were mostly related to local technical difficulties, such as the inability to find the site of a leak, or diffuse contamination of the abdomen. Postoperative complications classified according to Clavien–Dindo and length of hospital stay were better for a laparoscopic approach compared with the conversion group.

Our results are in accordance with Wright [22], who analysed the feasibility of laparoscopic reoperation for early postoperative complications following colorectal surgery: laparoscopic reoperation is equivalent to or better than open reoperation in terms of 30-day mortality and morbidity. This is probably due to the potential of laparoscopy to reduce the systemic stress response in patients requiring repeated surgery [23]. As assessed in the recent review from Halkias et al. [24] a key role is played by surgeons’ expertise; pooling data from 17 articles dated from 2007 to 2020, the authors concluded that re-operative laparoscopic colorectal surgery is safe when performed by experienced hands. Similarly, Al-Rashedy and colleagues [25], focusing on the role of re-laparoscopy in the management of early bariatric surgery complications, described the importance of the surgeon's experience in choosing laparoscopy as re-operative approach. Re-laparoscopy may allow to maintain the benefits of a minimally invasive approach also in the setting of postoperative complications. As already stated, laparotomy is associated with increased pain, prolonged ileus and increased risk of abdominal infection, so it should be employed only after a definitive diagnosis has been made or on a clear indication based on the patient clinical conditions.

The most important limitation of our study is linked to the long period of observation of the included cases: the introduction of new devices during the 24 years in which the sample is enrolled, do not allow to reach definitive conclusions.

From this point of view, our results show RL is safe and effective for management of complications following laparoscopic gastric surgery when performed by expert surgeons; on the other hand, further studies with a larger sample size and a randomized design are needed to define a gold standard treatment.

## Data Availability

From corresponding author upon request.
